# Genome‐wide profiling of mRNA and lncRNA expression in dengue fever and dengue hemorrhagic fever

**DOI:** 10.1002/2211-5463.12576

**Published:** 2019-02-02

**Authors:** Xiao‐Lan Zhong, Xiao‐Ming Liao, Fei Shen, Hai‐Jian Yu, Wen‐Sheng Yan, Yun‐Fang Zhang, Jia‐Jun Ye, Zhi‐Ping Lv

**Affiliations:** ^1^ Department of Quality Control Huadu Hospital of Southern Medical University & Guangzhou Huadu District People's Hospital China; ^2^ Department of Medicine Huadu Hospital of Southern Medical University & Guangzhou Huadu District People's Hospital China; ^3^ Clinical Laboratory Huadu Hospital of Southern Medical University & Guangzhou Huadu District People's Hospital China; ^4^ College of Traditional Chinese Medicine Southern Medical University Guangzhou China

**Keywords:** DEGs, DElncRNAs, dengue fever, dengue hemorrhagic fever, genome profiling, integrated analysis

## Abstract

Dengue fever (DF) and dengue hemorrhagic fever (DHF) are recurrent diseases that are widespread in the tropics. Here, we identified candidate genes associated with these diseases by performing integrated analyses of DF (GSE51808) and DHF (GSE18090) microarray datasets in the Gene Expression Omnibus (GEO). In all, we identified 7635 differentially expressed genes (DEGs) in DF and 8147 DEGs in DHF as compared to healthy controls (*P* < 0.05). In addition, we discovered 215 differentially expressed long non‐coding RNAs (DElncRNAs) in DF and 225 DElncRNAs in DHF. There were 1256 common DEGs and eight common DElncRNAs in DHF *vs* DF, DHF *vs* normal control, and DF *vs* normal control groups. Gene Ontology (GO) and Kyoto Encyclopedia of Genes and Genomes (KEGG) enrichment analysis revealed that signal transduction (false discovery rate = 8.33E‐10), ‘toxoplasmosis’, and ‘protein processing in endoplasmic reticulum’ were significantly enriched pathways for common DEGs. We conclude that the *MAGED1*,*STAT1*, and *IL12A* genes may play crucial roles in DF and DHF, and suggest that our findings may facilitate the identification of biomarkers and the development of new drug design strategies for DF and DHF treatment.

**Abbreviations**
AUC: area under the ROC curve
BP: biological process
DC: dendritic cell
DEG: differentially expressed gene
DElncRNA: differentially expressed long non‐coding RNA
DEmRNA: differentially expressed mRNA
DENV: dengue virus
DF: dengue fever
DHF: dengue hemorrhagic fever
ER: endoplasmic reticulum
FDR: false discovery rate
GEO: the Gene Expression Omnibus database
GO: Gene Ontology
IFN: interferon
lncRNA: long non‐coding RNA
KEGG: Kyoto Encyclopedia of Genes and Genomes
MAGED1: melanoma‐associated antigen D1
MF: molecular functions
NC: normal control
PPI: protein–protein interaction
ROC: receiver operating characteristic
STAT1: signal transducer and activator of transcription 1

Dengue fever (DF) is the second most severe infectious disease worldwide by mortality and morbidity after malaria 1. Clinically, DF is a mosquito‐borne illness that is caused by infection with dengue virus (DENV), especially affecting children in endemic, mostly tropical areas 2. In total, 33% of the world's population is at risk of infection with the DENV. The majority of DENV infections are symptomless or produce a slight illness with flu‐like symptoms, such as headache, fever, myalgia and decreased platelet counts and leucopenia. These symptoms are known as DF, which is an acute, self‐limited, febrile illness. However, some DF patients develop a severe syndrome known as dengue hemorrhagic fever (DHF), in which patients may display hematomas with marked thrombocytopenia or extremely low platelet counts 3. The clinical hallmark of DHF is plasma leakage, which usually lasts for approximately 48 h and leads to reduced circulatory volume 4.

During DENV infection, overproduction of cytokines and chemokines is considered to contribute to the increased vascular permeability, disruption of the coagulation system and shock associated with DHF 5. Despite its high burden on global health, no accessible antivirals or vaccines have been approved for clinical use 6. At present, there is no specific therapy available for DF and DHF. Appropriate fluid management to correct hypovolemia has been successful in reducing the mortality of DF and DHF 7, 8, but access to medical services remains problematic in many developing countries. Mosquito control, which is costly and often ineffective, still remains the only method of preventing DF and DHF currently available 2.

In this study, through integrated analysis, we aimed to obtain more accurate results with a large sample size than those through individual studies 9. In order to obtain the key long non‐coding RNAs (lncRNAs), mRNAs associated with DF and DHF, the study analyzed the blood transcriptome of DF patients, DHF patients and normal controls, seeking to identify early detection biomarkers of DF and DHF.

## Methods

### Eligible gene expression profiles of DF and DHF

We selected gene expression datasets of DF and DHF from the Gene Expression Omnibus database (GEO, http://www.ncbi.nlm.nih.gov/geo) which is the largest database of high‐throughput gene expression data 10. Search keywords were (‘dengue’ [MeSH Terms] OR ‘dengue fever’ [All Fields]) AND ‘Homo sapiens’ [porgn] AND ‘gse’ [Filter]. The datasets that met the following criteria were included in our study: (a) the selected dataset was genome‐wide mRNA transcriptome profiling by array; (b) the data were derived from DF or DHF patients; and (c) the datasets were normalized or raw datasets.

### Identification of common differentially expressed mRNAs and lncRNAs in the comparisons of DF *vs* normal control, DHF *vs* normal control, and DF *vs* DHF

Background correction was performed for the downloaded raw data. Using the limma package and the metama package, the inverse normal method was used for *P*‐value consolidation. The adopted standard of differential analysis was *P* < 0.05. Finally, the differentially expressed mRNAs (DEmRNAs) and differentially expressed long non‐coding RNAs (DElncRNAs) of DF *vs* normal control (NC), DHF *vs* NC and DF *vs* DHF were obtained 11. The Wilcoxon rank‐sum test was used to identify significant differences in the expression of mRNAs and lncRNAs between the different groups.

### Functional annotation of common differentially expressed genes

To identify the function and the potential pathways of common differentially expressed genes (DEGs), Gene Ontology (GO) classification (molecular functions, biological processes and cellular component) and Kyoto Encyclopedia of Genes and Genomes (KEGG) pathway enrichment were performed by using the online software genecodis3 ( http://genecodis.cnb.csic.es/analysis) 12. False discovery rate (FDR) < 0.05 was defined as the criterion of statistical significance.

### Protein–protein interaction network construction of common DEGs

To further research the biological functions of common DEGs, Cytoscape was used to search protein interaction of the top 100 up‐regulated common DEGs and top 100 down‐regulated common DEGs in the comparison of DHF *vs* DF in the BioGRID database. After removing non‐common genes, a protein–protein interaction (PPI) network was constructed 13, 14, 15. The network consisted of nodes and edges in which the nodes represent the proteins and the lines represent the interactions between them 16.

### Validation in the Gene Expression Omnibus dataset and receiver operating characteristic analysis

The GSE38246 dataset was obtained from the Gene Expression Omnibus (GEO; https://www.ncbi.nlm.nih.gov/geo/), and the amount of samples of normal:DF:DHF is 8:53:32. The expression pattern of selected DEmRNAs was verified using the GSE38246 dataset. In order to access the diagnostic value of DEmRNAs for DF, the ‘pROC’ package was used to calculate the receiver operating characteristic (ROC), and the area under the ROC curve (AUC) was further calculated. When the AUC value was > 0.6, the DEmRNAs were considered to be capable of distinguishing patients with DF from NC with excellent specificity and sensitivity.

## Results

### Differential expression analysis of genes in DF and DHF

The probes corresponding to multiple genes were removed, and for multiple probes corresponding to only one gene, the one with the largest average expression was retained. Finally, 18 756 genes were obtained for the differential analysis, of which 968 were lncRNAs and 17 788 were mRNAs according to the GRCh38.p7 reference genome.

**Figure 1:**
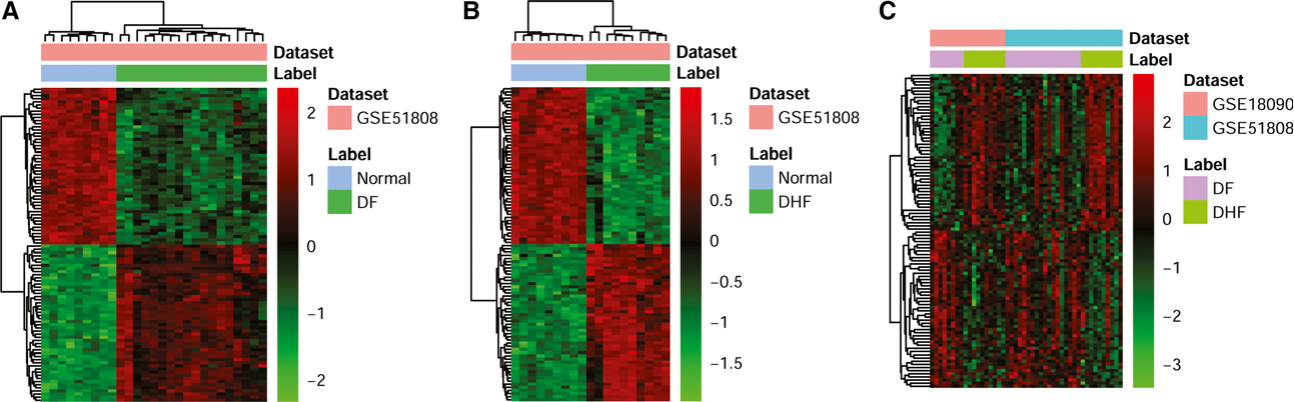
Heatmap image displaying genes that were significantly up‐regulated or down‐regulated (*P*‐value < 0.05) in DF and DHF compared to NC. (A) DF *vs* NC. (B) DHF *vs* NC. (C) DHF *vs* DF.

Compared with NC, 7635 DEGs in DF were obtained with *P* < 0.05, among which 4190 genes were up‐regulated and 3445 genes were down‐regulated. Likewise, 8147 DEGs in DHF were obtained, with 4239 up‐regulated and 3908 down‐regulated genes. The hierarchical clustering of the top 100 most significantly up‐ or down‐regulated genes was performed, and listed in the heatmap [Fig. 1A (DF) and Fig. 1B (DHF)]. Compared with the DF group, 2677 DEGs were obtained, among which, 1390 genes were up‐regulated and 1287 genes were down‐regulated in the DHF group. The top 100 most significantly up‐ or down‐regulated genes are listed in Fig. 1C.

### Differential expression analysis of lncRNAs in DF and DHF

**Figure 2:**
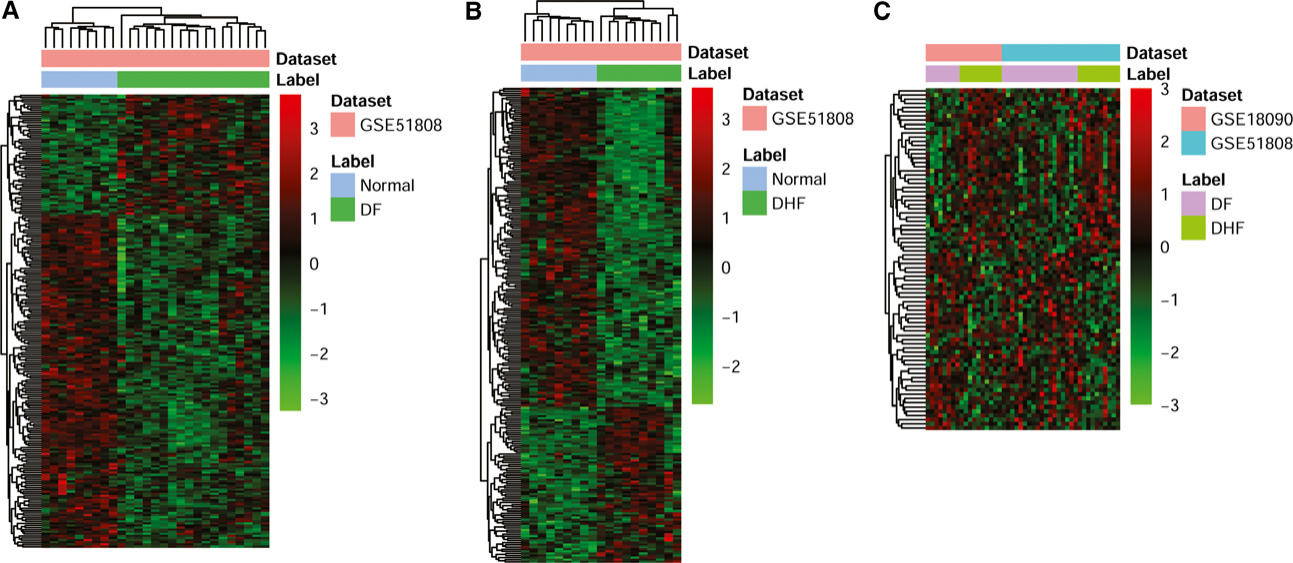
Heatmap image displaying lncRNAs that were significantly up‐regulated or down‐regulated (*P*‐value < 0.05) in DF and DHF compared to NC. (A) DF *vs* NC. (B) DHF *vs* NC. (C) DHF *vs* DF.

Compared with NC, 215 DElncRNAs in DF were obtained with *P* < 0.05, among which, 57 lncRNAs were up‐regulated and 158 lncRNAs were down‐regulated. Likewise, of 225 DElncRNAs in DHF, 74 up‐regulated and 151 down‐regulated lncRNAs were obtained [Fig. 2A (DF) and Fig. 2B (DHF)]. Compared with the DF group, there were 81 DElncRNAs, among which, 42 DElncRNAs were up‐regulated and 39 DElncRNAs were down‐regulated in DHF group (Fig. 2C).

### Common DEGs and lncRNAs of DHF *vs* DF, DHF *vs* NC and DF *vs* NC

**Figure 3:**
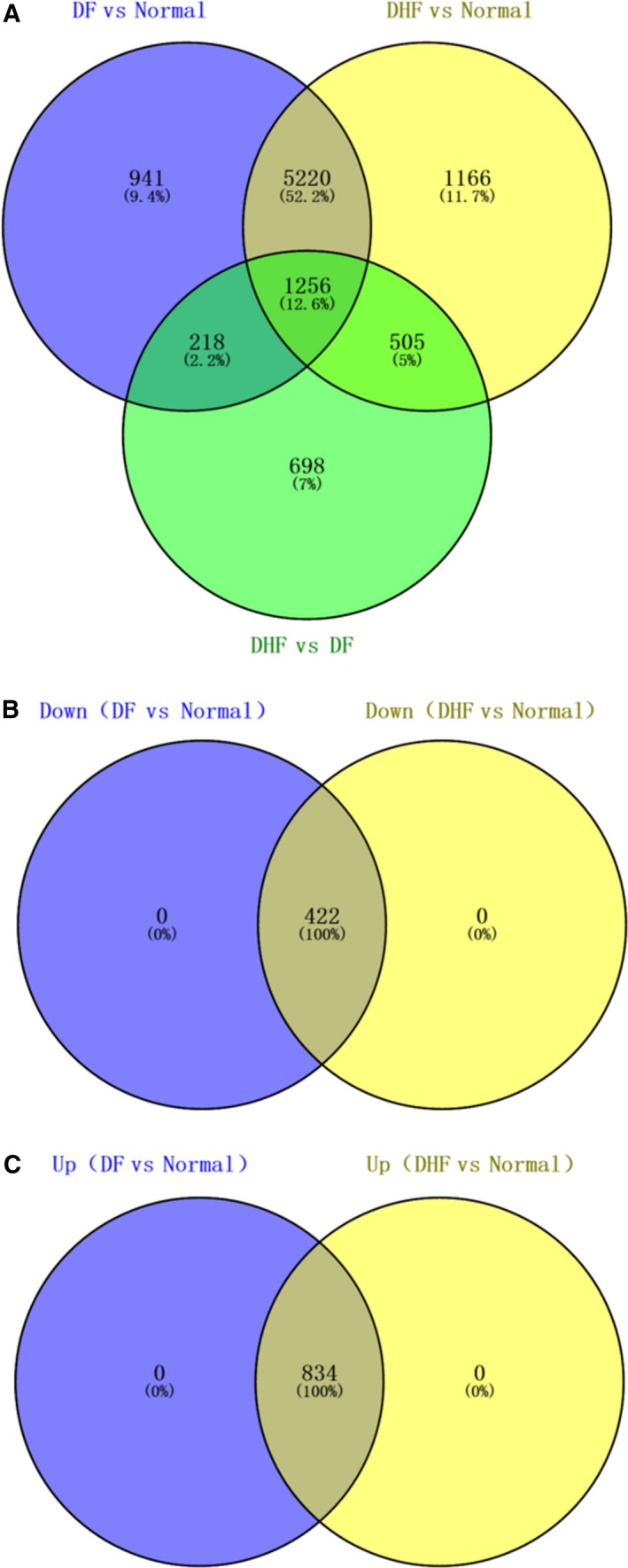
Venn diagram showing the overlap of differentially expressed mRNAs in DF *vs* NC, DHF *vs* NC and DF *vs* DHF. Numbers represent the number of DEmRNAs; percentages represent the ratio of current DEmRNAs to total.

**Figure 4:**
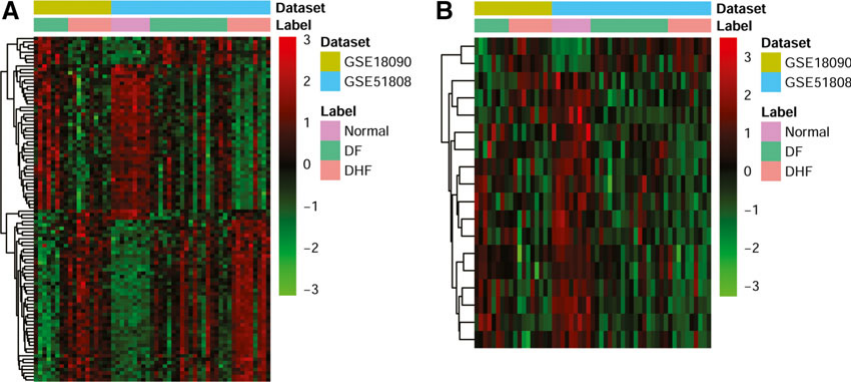
Heatmap image displaying common genes and lncRNAs that were significantly up‐regulated or down‐regulated (*P*‐value < 0.05) in DF *vs* NC, DHF *vs* NC, DHF *vs* DF. (A) mRNA. (B) lncRNAs).

In Fig. 3, there were 1256 common DEGs in DHF *vs* DF, DHF *vs* NC and DF *vs* NC, of which 834 DEGs were up‐regulated, and 422 DEGs were down‐regulated in DF or DHF compared to NC. A hierarchical clustering heatmap for the top 100 (DHF *vs* DF) common genes is shown in Fig. 4A. A total of 18 common DElncRNAs were obtained in DHF *vs* DF, DHF *vs* NC and DF *vs* NC, of which 16 DElncRNAs were down‐regulated and two DElncRNAs were up‐regulated in DF or DHF compared to NC. The hierarchical clustering heatmap of all common lncRNAs is shown in Fig. 4B.

### Functional annotation of common DEGs

**Figure 5:**
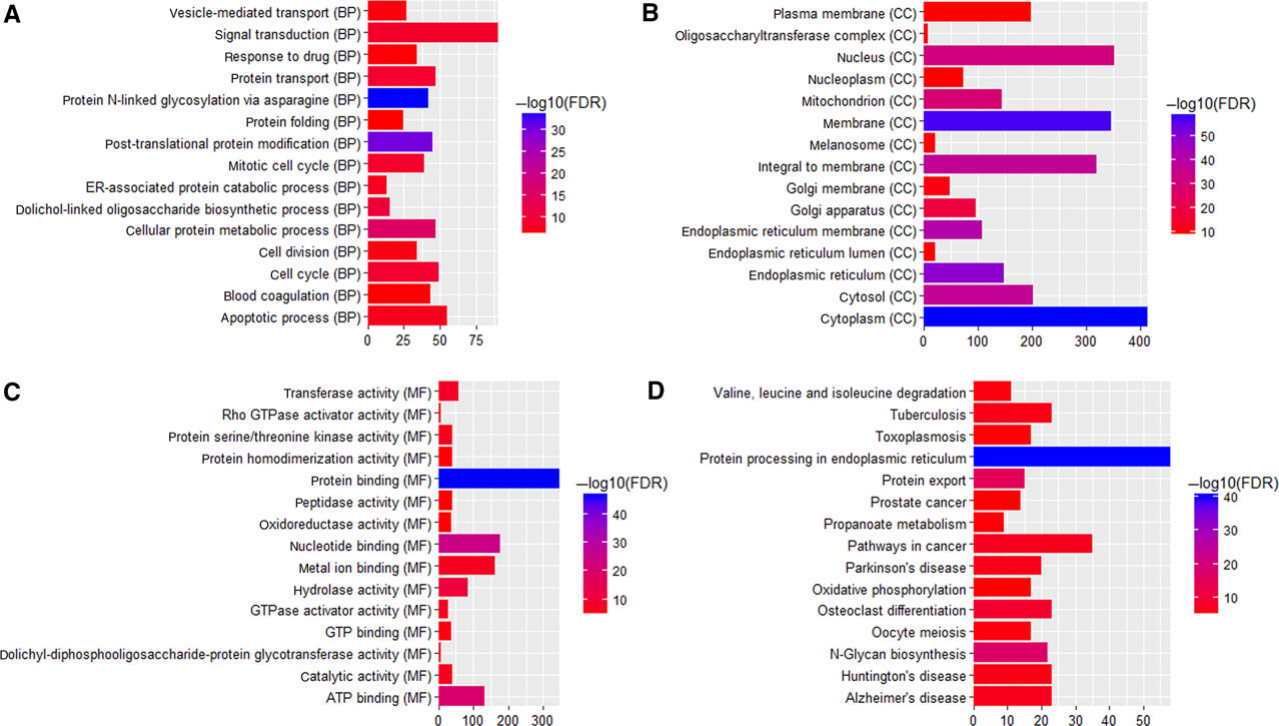
GO pathway analyses of dysregulated protein‐coding genes. (A) Biological process; (B) cellular component; (C) molecular functions; (D) KEGG.

A total of 1256 common DEmRNAs were subjected to GO enrichment and KEGG enrichment analysis using the R language (gseabase package). GO enrichment analysis and KEGG pathway analysis indicated that these common DEGs were significantly involved in the biological processes of signal transduction (FDR = 8.33E‐10), apoptotic process (FDR = 1.46E‐08), cell cycle (FDR = 3.04E‐10) and protein transport (FDR = 5.19E‐10) (Fig. 5A). In addition, cytoplasm (FDR = 3.19E‐60), membrane (FDR = 7.16E‐58) and nucleus (FDR = 3.37E‐32) were remarkably enriched cytology components (Fig. 5B), and protein binding (FDR = 3.37E‐32), nucleotide binding (FDR = 3.58E‐25) and metal ion binding (FDR = 7.45E‐08) were significantly involved molecular functions (Fig. 5C). Protein processing in endoplasmic reticulum (FDR = 6.36E‐41), N‐glycan biosynthesis (FDR = 1.44E‐17) and toxoplasmosis (FDR = 2.14E‐05) were significant enriched KEGG pathways (Fig. 5D).

### PPI network and module analysis of common DEGs

**Figure 6:**
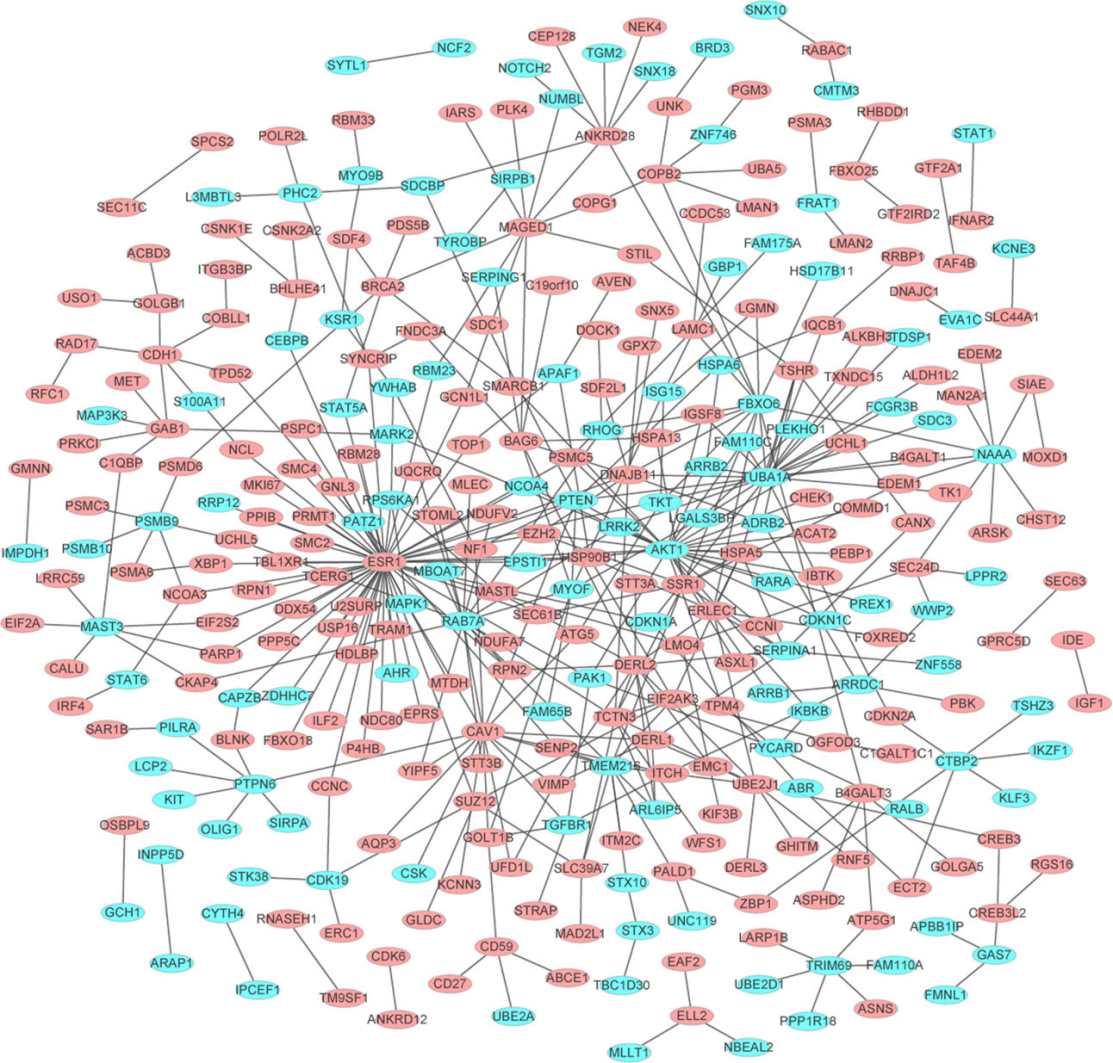
Protein–protein interaction network of common genes. All points are differentially expressed genes; green represents down‐regulated and red represents up‐regulated.

To identify potential interactions among common DEGs, a PPI network was constructed. The PPI results identified 330 nodes (genes) and 419 edges (Fig. 6: all points are proteins encoded by common DEGs, a green oval indicates proteins encoded by a down‐regulated DEG (DHF *vs* DF) and a red oval indicates proteins encoded by an up‐regulated DEG (DHF *vs* DF). Among them, those of higher degree are ESR1 (degree = 57), AKT1 (degree = 29), TUBA1A (degree = 23), CAV1 (degree = 17), RAB7A (degree = 17), FBXO6 (degree = 14), DERL2 (degree = 1), TMEM216 (degree = 11), MAGED1 (degree = 10) and DNAJB11 (degree = 10).

### Proximity analysis of DElncRNA‐DEmRNA

Three pairs of DElncRNA–adjacent DEG (including two lncRNAs and three DEGs) were obtained by searching 100 kb upstream and downstream of common DElncRNAs. The expression of common DElncRNAs and adjacent DEGs is listed in Table 1.

**Table 1:** Differential expression of common lncRNAs and adjacent differentially expressed common genes

	LncRNA			Nearby mRNA		
chr	Symbol	Start − 100 kb	End + 100 kb	Symbol	Start	End
9	LOC100129034	124253473	124459186	*NEK6*	124257606	124353307
17	TNRC6C‐AS1	78007398	78211799	*AFMID*	78187317	78207701
17	TNRC6C‐AS1	78007398	78211799	*TK1*	78174075	78187233

### Validation of the expression of DEmRNAs by GSE38246

**Figure 7:**
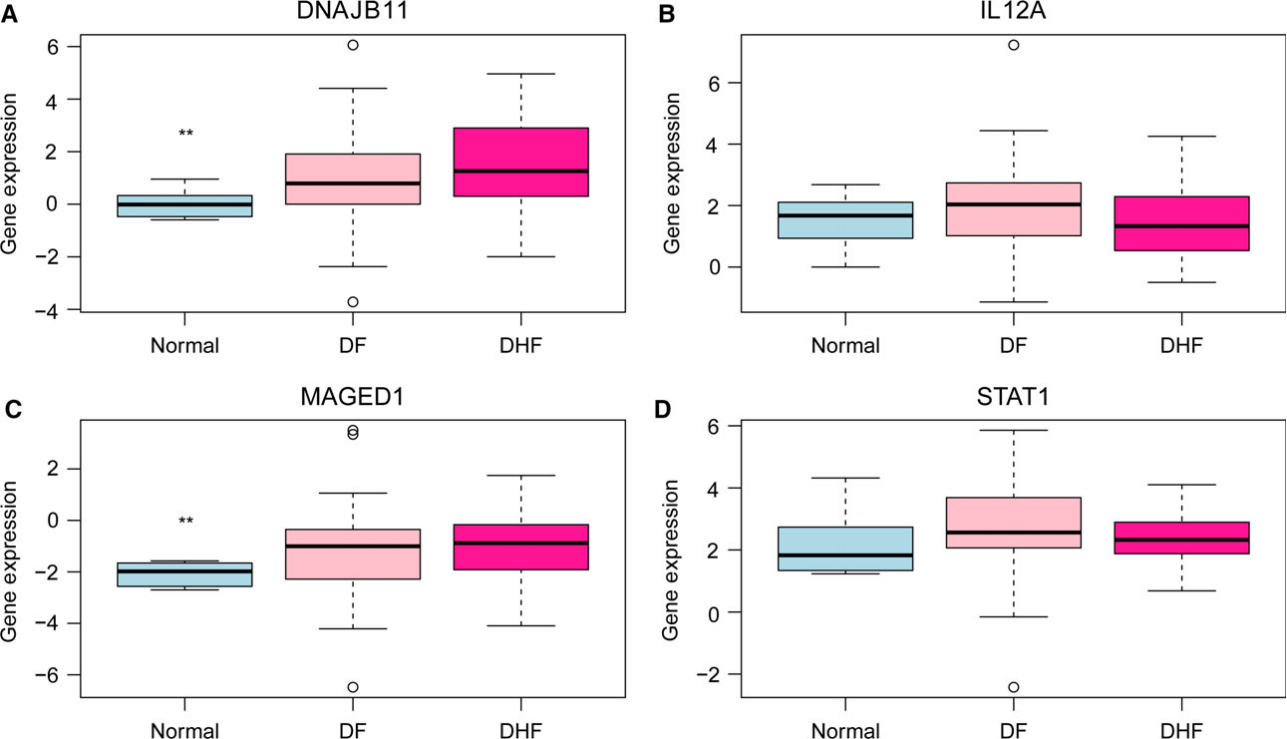
Validation of the expression levels of selected DEGs in DF and DHF based on GSE38246 The *x*‐axis shows case and normal groups and *y*‐axis shows gene expression level. (A) *DNAJB11*; (B) *IL12A*; (C) *MAGED1*; (D) *STAT1*.

Based on GSE38246, expression of four DEmRNAs (*DNAJB11*,* IL12A*,* MAGED1*, and *STAT1*) was validated (Fig. 7). Expression of three DEmRNAs (*DNAJB11*,* IL12A*, and *MAGED1*) was up‐regulated in DF and DHF compared to NC. These results were generally consistent with the results of our integrated analysis.

### ROC curve analysis

**Figure 8:**
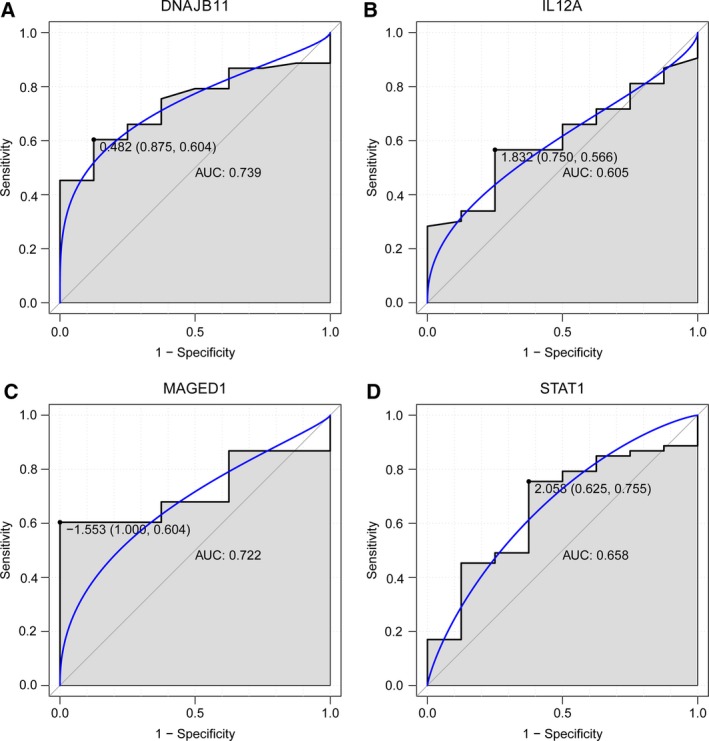
The ROC curves of selected DEGs between DF patients and healthy controls. The ROC curves were used to show the diagnostic ability of these selected DEGs with sensitivity and specificity. The *x*‐axis shows 1 − specificity and *y*‐axis shows sensitivity. (A) *DNAJB11*; (B) *IL12A*; (C) *MAGED1*; (D) *STAT1*.

ROC curve analyses and the AUC were used to assess the discriminatory ability of four DEmRNAs (*DNAJB11*,* IL12A*,* MAGED1*, and *STAT1*). The AUCs of all these four DEmRNAs, including *DNAJB11* (0739), *IL12A* (0.605), *MAGED1* (0.722) and *STAT1* (0.658), were more than 0.6 (Fig. 8), which had great diagnostic value for DF.

## Discussion

In severe cases, DENV, which is an alarming emerging disease, can be fatal. The activation of multiple inflammatory pathways is involved in the pathogenesis of severe critical disease following DENV infection. The response to DENV infection is complicated and characterized by the production of numerous cytokines 9. In our study, we aimed to identify the gene and lncRNA expression profiles of DF or DHF. We found 1256 common DEGs in the comparisons of DHF *vs* DF, DHF *vs* NC and DF *vs* NC groups, and interestingly the expression trend of these common genes was essentially identical for both DF and DHF patients compared to the NC samples. A total of 18 common DElncRNAs were obtained, of which 16 DElncRNAs were down‐regulated, two DElncRNAs were up‐regulated (DF *vs* NC and DHF *vs* NC). These indicated that DF and DHF may have similar features. For the common DEGs and DElncRNAs, we also constructed the PPI network, functional annotation and adjacent analysis of mRNAs and lncRNAs. Together with retrieved literature, we obtained three genes that may be involved in DF and DHF, namely *MAGED1*,* STAT1*, and *IL12A*. These three genes were among 1256 common differentially expressed genes in DHF *vs* DF, DHF *vs* NC and DF *vs* NC.

MAGED1 (melanoma‐associated antigen D1, also known as NRAGE or Dlxin‐1) is a member of the MAGE homology domain (MHD)‐containing protein superfamily, which includes over 30 members in humans 17. The possibility that they are candidates for disease is raised by the strong expression of the *MAGED1* genes in structures involved in higher function, such as the cerebral cortex, and the hippocampus 18. *MAGED1* is highly expressed throughout the brain 18. Previous study has found that during the acute phase of dengue, the expression level of *MAGED1* was greater in DHF patients compared to DF patients 19. Interestingly, in patients of dengue and the acute DHF patients, the pro‐apoptotic *PDRX4* and *MAGED1* genes were over‐expressed. *MAGED1* has been associated with the p75 neurotrophin receptor‐mediated programmed cell death pathway 20. Indeed, DENV infection augments apoptosis in patients with severe DHF, so the expression of pro‐apoptotic genes, such as *MAGED1*, was increased 19. In our study, *MAGED1* expression was up‐regulated in patients with DF and DHF compared with NC. In the PPI network, *MAGED1* was among the top 10 genes of higher degree. All of these results indicated that *MAGED1* may play a role in the pathogenesis of DF and DHF.

IL12A/IL‐12p35, which is a subunit of a cytokine, acts on T and natural killer cells and has a broad array of biological activities. *IL12A* is crucial for the T‐cell‐independent induction of IFN‐γ, and is required for the differentiation of both Th1 and Th2 cells. The responses of lymphocytes to this cytokine are mediated by the activator of transcription protein STAT4 21. In the study of de Kruif, the profile showed characteristics of a general antiviral response with up‐regulation of *IL12A*, which is a potent stimulator of IFN‐γ production 9.

Signal transducer and activator of transcription 1 (STAT1), which is encoded by the *STAT1* gene, is a transcription factor in humans. STAT1 is a member of the STAT protein family. A recent study of a panel of 184 inflammation‐related genes showed that the *STAT1* gene was one of the most differentially expressed 22. In the study of Cerny, nanostring gene expression data showed significant up‐regulation of *STAT1* upon dengue viral exposure in susceptible dendritic cell populations 23. Yu *et al*. demonstrated that STAT1‐mediated antiviral interferon responses contribute to the action of schisandrin A against DENV replication 24. In our study, the *STAT1* expression was up‐regulated in DF *vs* NC and DHF *vs* NC, and was down‐regulated in DHF *vs* DF.

In our functional annotation, we found that DEGs were significantly involved in the biological processes of signal transduction. Recent studies have shown that DENV can induce apoptosis 25, programmed cell death can be observed in endothelial cells, hepatocytes, neuroblastoma cells and hepatoma cells 26, and its signaling and transduction pathways have been studied in great depth. However, it is reasonable to speculate that the capsid protein can participate in the signal transduction of host cells, cause apoptosis of the host cells, and lead to the development of the disease. In the KEGG results, we found that DF‐related genes, *STAT1* and *IL12A*, were enriched in the signaling pathway of ‘toxoplasmosis’, and *MAGED1* was enriched in ‘protein processing in endoplasmic reticulum’. This may indicate that there were some similar features in the pathogenic mechanism of DF and DHF compared with toxoplasmosis. As previously described, endoplasmic reticulum (ER) rearrangement and expansion is an early event in the DENV life cycle that is driven by viral but not host protein synthesis 27. Reid *et al*. 28 reported that throughout the viral life cycle, DENV plus‐ and minus‐strand RNAs were highly partitioned to the ER, identifying the ER as the primary site of DENV translation. The KEGG enrichment analysis in the present study supported the previous studies and suggested that ‘toxoplasmosis’ and ‘protein processing in endoplasmic reticulum’ pathways are involved with DF and DHF.

## Conclusion

This study provides further insight into the molecular aspects of DF and DHF, suggesting new molecular signatures and new targets for development of specific biomarkers. In particular, our findings suggest the possibility that signal transduction‐related genes may also be factors indicating a poor prognosis for DF and DHF.

## Conflict of interest

The authors declare no conflict of interest.

## Author contributions

XL and XZ drafted the manuscript; FS and HY participated in clinical data and collection; YZ and WY carried out the data analysis; ZL and JY had significant roles in the study design and manuscript review. All authors read and approved the final manuscript.
